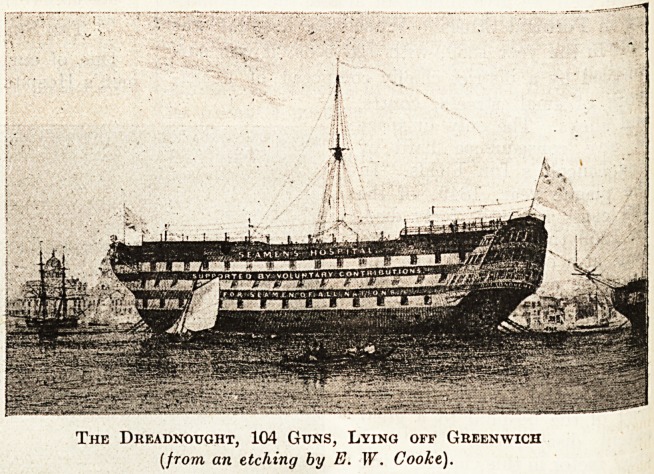# As Depicted by Engraved Views.—IV

**Published:** 1921-01-15

**Authors:** 


### THE HISTORY OF HOSPITALS

### AS DEPICTED BY ENGRAVED VIEWS.—IV.

ONE of the first hospitals to be founded in the nineteenth century was King's College, and the small view we give affords an idea of the old building in Portugal Street. When the hospital was built in the year 1839, with 120 beds, it was surrounded by a district chiefly composed of a network of small streets, courts, and alleys. The only air-space of any consequence hard by was Lincoln's Inn Fields. In the plague year, 1849, of the 123 cases received, forty died. In 1859 the hospital was rebuilt at a cost of £50,000, and from the day of opening until July 15, 1913, its doors were never closed to a deserving case.

But, during the seventy-four years of splendid service in West Central London, the district of the hospital to a great extent disappeared, the first chief factor in this change being the building of the Law Courts, which removed from the map such slums as Chair Court, Pickett Place, and Ship and Anchor Court. In one of these rookeries there were six small houses tenanted by forty-eight families, which included, besides adults, 158 children. The important improvement caused by the formation of Kingsway and Aldwych was the second factor, and the Committee of Management decided to move the work to Denmark Hill, close to such a populous locality as Camberwell.

KING'S COLLEGE HOSPITAL, PORTUGAL STREET, ABOUT 1840 (*from an old print*).

### THE SEAMEN'S HOSPITAL, GREENWICH.

One of our most romantic hospitals is the Seamen's Hospital at Greenwich, where at almost any time may be found sailors of every colour and tongue, stricken by accident or illness on entering the Port of London. Of the present building, once the infirmary of Greenwich Hospital, but now transformed and improved so greatly as to become one of our most efficient institutions, there are, we

SITE OF THE NEW KING'S COLLEGE HOSPITAL 100 YEARS AGO.

Previous articles appeared on Nov. 13, Dec. 4 and 18, 1920

**The History of Hospitals**—(*continued*).

believe, few, if any, engraved views. But of the "Dreadnought" ship,. when lying in the river, there are many illustrations, for naturally a permanent floating hospital is a unique thing which justifies pictorial perpetuation. In the winter of 1817-18 the first hospital of this great Society was founded on board the "Grampus" (a fifty-gun ship), moored off Greenwich. This vessel being found too small for the purpose, the Government exchanged it in 1830 for the 104 - gun ship "Dreadnought" (see illustration). After twenty-seven years' service she became unhealthy, and was replaced by a still larger vessel, H.M.S. "Caledonia," 120 guns, which was renamed "Dreadnought."

THE DREADNOUGHT, 104 GUNS, LYING OFF GREENWICH (*from an etching by E. W. Cooke*).

THE MIDDLESEX.

We reproduce an old view of the Middlesex Hospital, founded in 1745, and standing, according to an old account, "in Mary-bone Fields, near Oxford Road, now Charles Street." Our view (about 1775) mainly corresponds with an earlier print of 1755, except that the surrounding wall has been beautified and provided with several extra doors. Later views show the coming and passing of a belfry over the central entrance, and the complete disappearance of the enclosing wall. The Middlesex, as its history in engraved views shows, is one of those institutions which has been altered and enlarged, yet, from the aspect best known to the

MIDDLESEX HOSPITAL, ABOUT 1775 (*from an old print*).

public, has retained the chief architectural characteristics of its original building.

As was stated in the first article of this series, by far the most of the engravings of hospitals will be found in topographical books published early last century or late in the century before, in which the letterpress is generally of a less important nature than the illustrations. Sometimes the same plates were used in more than one book, a new publisher having bought the stock-in-trade and presented the illustrations again in a different grouping. Many of these volumes have been broken up, and the collector may come across the engravings sold singly in the portfolios of book and curiosity shops in Charing Cross Road and some of its side-streets, in thoroughfares near Lincoln's Inn Fields and Red Lion Square, and other places.

Amongst some of the better-known topographical works may be mentioned Shepherd's "London in the Nineteenth Century (1829), in two quarto volumes, which are full of fine engravings from steel plates after the design of Thomas H. Shepherd. Others are "Ackermann's Microcosm of London," and the other issues of the same publishing house. The "Microcosm" came out in parts, which, when bound, make three quarto volumes. The architectural details of the plates are by Pugin, and the figures are filled in by Rowlandson, often in a very characteristic manner. In the letterpress we learn that the hospitals in London at that date (about 1805) were St. Thomas's ("for sick and lame, especially sailors"), "Gray's" (an obvious misprint for "Guy's"), the London, St. George's, Westminster General Infirmary, St. Bartholomew's, the Lock Hospital, Bethlem, St. Luke's, Middlesex, six unnamed lying-in hospitals, and two hospitals that have disappeared—Hospital Misericordia (for venereal diseases), in Goodman's Fields, and the Smallpox Hospital, St. Pancras.

## Figures and Tables

**Figure f1:**
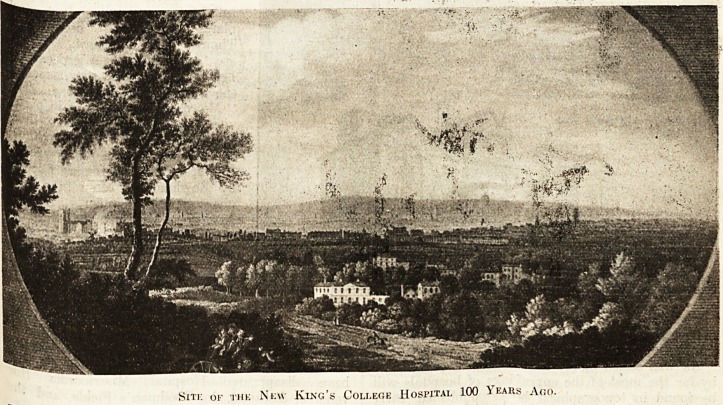


**Figure f2:**
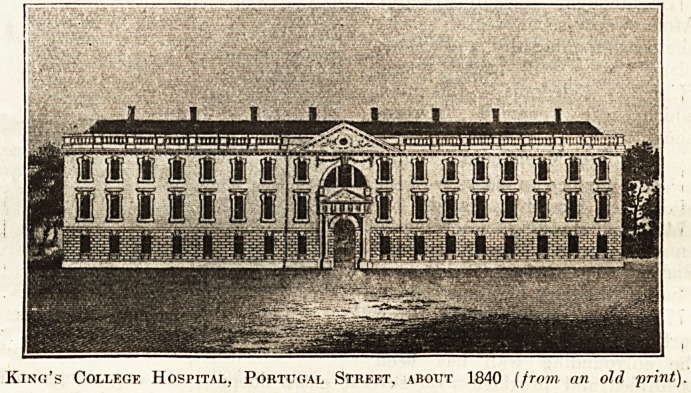


**Figure f3:**
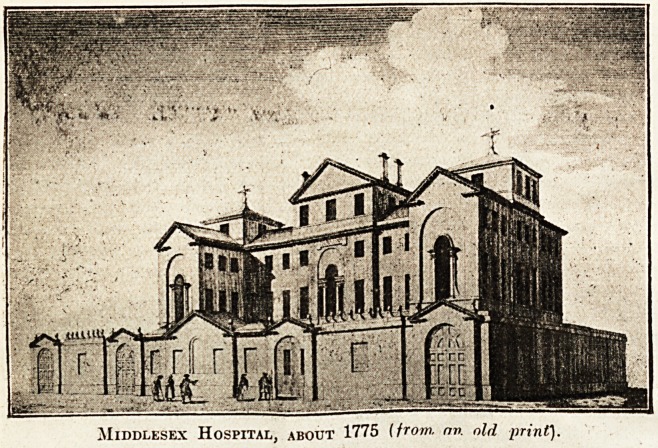


**Figure f4:**